# Dbf4-Dependent Kinase (DDK)-Mediated Proteolysis of CENP-A Prevents Mislocalization of CENP-A in *Saccharomyces cerevisiae*

**DOI:** 10.1534/g3.120.401131

**Published:** 2020-04-15

**Authors:** Jessica R. Eisenstatt, Lars Boeckmann, Wei-Chun Au, Valerie Garcia, Levi Bursch, Josefina Ocampo, Michael Costanzo, Michael Weinreich, Robert A. Sclafani, Anastasia Baryshnikova, Chad L. Myers, Charles Boone, David J. Clark, Richard Baker, Munira A. Basrai

**Affiliations:** * Genetics Branch, Center for Cancer Research, National Cancer Institute,; † Division of Developmental Biology, Eunice Kennedy Shriver National Institute for Child Health and Human Development, National Institutes of Health, Bethesda, Maryland 20894,; ‡ Department of Molecular Genetics,; § Donnelly Centre for Cellular and Biomolecular Research, Toronto, Ontario M5S 3E1, Canada,; ** Van Andel Research Institute, 333 Bostwick Ave NE, Grand Rapids, MI 49503,; †† Department of Biochemistry and Molecular Genetics, University of Colorado School of Medicine, Aurora, Colorado 80045,; ‡‡ Lewis-Sigler Institute for Integrative Genomics, Princeton University, Princeton, New Jersey 08544,; §§ Department of Computer Science and Engineering, University of Minnesota-Twin Cities, Minneapolis, Minnesota 55455, and; *** Department of Microbiology and Physiological Systems, University of Massachusetts Medical School, Worcester, Massachusetts 01655

**Keywords:** Centromere, Cse4, CENP-A, DDK, Psh1, Cdc7

## Abstract

The evolutionarily conserved centromeric histone H3 variant (Cse4 in budding yeast, CENP-A in humans) is essential for faithful chromosome segregation. Mislocalization of CENP-A to non-centromeric chromatin contributes to chromosomal instability (CIN) in yeast, fly, and human cells and CENP-A is highly expressed and mislocalized in cancers. Defining mechanisms that prevent mislocalization of CENP-A is an area of active investigation. Ubiquitin-mediated proteolysis of overexpressed Cse4 (*GALCSE4*) by E3 ubiquitin ligases such as Psh1 prevents mislocalization of Cse4, and *psh1**Δ* strains display synthetic dosage lethality (SDL) with *GALCSE4*. We previously performed a genome-wide screen and identified five alleles of *CDC7* and *DBF4* that encode the Dbf4-dependent kinase (DDK) complex, which regulates DNA replication initiation, among the top twelve hits that displayed SDL with *GALCSE4*. We determined that *cdc7**-7* strains exhibit defects in ubiquitin-mediated proteolysis of Cse4 and show mislocalization of Cse4. Mutation of *MCM5* (*mcm5**-bob1*) bypasses the requirement of Cdc7 for replication initiation and rescues replication defects in a *cdc7**-7* strain. We determined that *mcm5**-bob1* does not rescue the SDL and defects in proteolysis of *GALCSE4* in a *cdc7**-7* strain, suggesting a DNA replication-independent role for Cdc7 in Cse4 proteolysis. The SDL phenotype, defects in ubiquitin-mediated proteolysis, and the mislocalization pattern of Cse4 in a *cdc7**-7 **psh1**Δ* strain were similar to that of *cdc7**-7* and *psh1**Δ* strains, suggesting that Cdc7 regulates Cse4 in a pathway that overlaps with Psh1. Our results define a DNA replication initiation-independent role of DDK as a regulator of Psh1-mediated proteolysis of Cse4 to prevent mislocalization of Cse4.

The centromere, a specialized region of the chromosome that is essential for faithful chromosome segregation, and associated proteins make up the kinetochore, which serves as an attachment site for microtubules to promote segregation of sister chromatids during mitosis (Allshire and Karpen 2008; Verdaasdonk and Bloom 2011; Burrack and Berman 2012; Choy *et al.* 2012; Maddox *et al.* 2012; Mckinley and Cheeseman 2016). Budding yeast “point centromeres” consist of approximately 125 base pairs (bp) of unique DNA sequences, whereas other eukaryotic organisms have “regional centromeres” consisting of several mega-bp of repeated DNA sequences, satellite DNA arrays, or retrotransposon-derived sequences. Despite the difference in the size of centromeres, the centromeric histone H3 variant (Cse4 in *Saccharomyces cerevisiae*, Cnp1 in *Schizosaccharomyces pombe*, CID in *Drosophila melanogaster*, and CENP-A in mammals) is evolutionarily conserved from yeast to human cells and is essential for faithful chromosome segregation (Przewloka and Glover 2009; Choy *et al.* 2012; Henikoff and Furuyama 2012; Biggins 2013; Wong *et al.* 2020). Mislocalization of overexpressed CENP-A and its homologs to non-centromeric regions contributes to chromosomal instability (CIN) in yeast, fly, and human cells (Heun *et al.* 2006; Au *et al.* 2008; Mishra *et al.* 2011; Lacoste *et al.* 2014; Athwal *et al.* 2015; Shrestha *et al.* 2017). CIN and high expression of CENP-A have been observed in cancer cells and this correlates with poor prognosis and increased invasiveness (Tomonaga *et al.* 2003; Amato *et al.* 2009; Li *et al.* 2011; Mcgovern *et al.* 2012; Sun *et al.* 2016; Zhang *et al.* 2016). The mechanisms that prevent the mislocalization of CENP-A and its homologs are not fully understood. Defining these mechanisms will provide insight into how mislocalization of CENP-A contributes to aneuploidy in human cancers.

Stringent regulation of cellular levels of Cse4 by post-translational modifications such as ubiquitination prevents its mislocalization to non-centromeric regions in budding yeast, fission yeast, and flies (Collins *et al.* 2004; Moreno-Moreno *et al.* 2006; Moreno-Moreno *et al.* 2011; Au *et al.* 2013; Gonzalez *et al.* 2014). In addition to ubiquitination of Cse4, we have recently defined a role for sumoylation in proteolysis of Cse4 (Ohkuni *et al.* 2016). Multiple ubiquitin ligases, such as Psh1, Ubr1, the Sumo-targeted ubiquitin ligase Slx5, and the F-box protein Rcy1 regulate proteolysis of overexpressed Cse4 (Hewawasam *et al.* 2010; Ranjitkar *et al.* 2010; Cheng *et al.* 2016; Ohkuni *et al.* 2016; Cheng *et al.* 2017; Ohkuni *et al.* 2018). Psh1 is one of the best characterized E3 ligases for proteolysis of overexpressed Cse4 and prevents mislocalization of Cse4 to non-centromeric regions (Hewawasam *et al.* 2010; Ranjitkar *et al.* 2010). Psh1 interacts with the CENP-A targeting domain (CATD) in the C-terminus of Cse4 (Hewawasam *et al.* 2010; Ranjitkar *et al.* 2010) and mediates Cse4 degradation through the interaction of Psh1 with Spt16, a component of the FACT (facilitates chromatin transcription) complex (Deyter and Biggins 2014). It has also been shown that phosphorylation of Psh1 by casein kinase 2 (CK2) promotes degradation of Cse4 (Hewawasam *et al.* 2014). In addition to targeting the C-terminus of Cse4, we have shown that the N-terminus of Cse4 regulates Cse4 proteolysis (Au *et al.* 2013).

Mutant strains that show defects in Cse4 proteolysis display synthetic dosage lethality (SDL) when Cse4 is overexpressed. However, Cse4 is not completely stabilized in *psh1**Δ*, *ubr1**Δ*, *doa1**Δ*, *slx5**Δ*, or *rcy1**Δ* strains (Cheng *et al.* 2017), suggesting the existence of additional genes/pathways that regulate Cse4 proteolysis. We previously performed a Synthetic Genetic Array (SGA) using conditional mutant alleles of essential genes to identify additional factors that regulate Cse4 proteolysis (Au *et al.* 2020). The screen identified mutants encoding the F-box proteins Met30 and Cdc4 of the Skp1, Cullin, F-box (SCF) complex. We defined a cooperative role for Met30 and Cdc4 in the proteolysis of endogenous Cse4 to prevent its mislocalization and promote chromosome stability (Au *et al.* 2020). Here, we pursued studies of the evolutionarily conserved Dbf4-dependent kinase (DDK) complex as we identified five mutant *dbf4* and *cdc7* alleles among the top twelve significant SDL hits. The DDK complex, which is essential for the initiation of DNA replication, consists of the Cdc7 kinase and the regulatory subunit Dbf4 (Jackson *et al.* 1993; Stillman 1996). DDK promotes the initiation of DNA replication by phosphorylating Cdc45 and subunits of the mini-chromosome maintenance complex (*Mcm2*-7) at origins of replication (Lei *et al.* 1997; Owens *et al.* 1997; Zou and Stillman 2000; Bruck and Kaplan 2009). DDK also phosphorylates histone H3 at threonine 45 (H3T45) during S-phase, which occurs in response to replication stress (Baker *et al.* 2010), suggesting that H3T45 phosphorylation is linked with DNA replication. Previous studies have shown that centromeric association of Cdc7 is important for early replication of centromeres (Raghuraman *et al.* 2001; Rossbach *et al.* 2017), which are among the earliest firing origins.

The identification of five *cdc7* and *dbf4* alleles that display SDL with overexpressed Cse4 led us to investigate the role of DDK in regulating Cse4 proteolysis. We determined that Cdc7 regulates Cse4 proteolysis in a pathway that overlaps with Psh1, and this prevents mislocalization of Cse4. The role of Cdc7 in Cse4 proteolysis is independent of its role in the initiation of DNA replication.

## Material And Methods

### Strains and plasmids

Yeast strains were grown in YPD (1% yeast extract, 2% bacto-peptone, 2% glucose) or synthetic medium with glucose or raffinose/galactose (2% final concentration each) and supplements to allow for selection of the indicated plasmids. Yeast strains and plasmids used in this study are described in Table S1 and Table S2, respectively. To integrate the *cdc7**-7* allele marked with the G418 resistance marker (KanMX), the *cdc7**-7* sequence amplified from RSY302 and the KanMX sequence were cloned into pGEM-T-Easy. *cdc7**-7:KanMX* from the vector was transformed into yeast strains as per standard lithium acetate procedure. Transformants were screened for temperature sensitivity at 37° and sequenced (CCR Genomics Core) to confirm the G1137A mutation. Wild type *CDC7* marked with G418 resistance strains were selected from the non-temperature sensitive transformants and sequenced to verify the wild type *CDC7* sequence. To replace endogenous *CSE4* with HA-tagged *CSE4*, a PCR-based method was used as described previously (Boeckmann *et al.* 2013). Replacement of the *CSE4* gene with HA-tagged *CSE4* was verified by sequencing and Western blots confirmed the expression of the HA-tagged protein. At least two independent strains were analyzed for each experiment.

### Growth assays

Wild type and mutant strains were transformed with the indicated plasmids or the empty vector. Transformants grown on synthetic medium, selective for the plasmid, were suspended in water to a concentration with an optical density of 1 measured at a wavelength of 600 nm (OD_600_, approximately 1.0 X 10^7^ cells per ml). Fivefold serial dilutions starting with 1 OD_600_ were generated and 3 μl of each dilution spotted on synthetic growth medium selecting for the plasmid and containing either glucose (2% final concentration) or galactose and raffinose (2% final concentration each). Strains were grown at the indicated temperatures for 3-5 days. Three independent transformants were assayed for growth unless otherwise stated.

### Protein stability assays

Protein stability assays were performed as previously described (Au *et al.* 2008). Briefly, strains were grown to logarithmic phase overnight in selective media, re-suspended in fresh media containing galactose/raffinose (2% final concentration each) and grown for 1.75 or four hours as indicated in figure legends at 23°. 10 µg/ml cycloheximide (CHX) and glucose (2% final concentration) were added to cultures and aliquots were collected 0, 30, 60, 90, and 120 min after CHX addition. Proteins were isolated using the TCA method as described previously (Kastenmayer *et al.* 2006). Protein levels were standardized using the Bio-Rad DC Protein Assay. Samples were diluted 1:1 with Laemmli buffer containing BME and stored at -20° for Western blot analysis. Proteins were separated by SDS-PAGE on 4–12% Bis-TRIS SDS-polyacrylamide gels (Novex, NP0322BOX). Western blot analysis was done using primary antibodies α-HA (1:1000, Roche, 12CA5), α-Flag (1:5000, Sigma, F3165), or α-Tub2 (1:4500, custom made for Basrai Laboratory) in TBST containing 5% (w/v) dried skim milk. HRP-conjugated sheep α-mouse IgG (Amersham Biosciences, NA931V) and HRP-conjugated donkey α-rabbit IgG (Amersham Biosciences, NA934V) were used as secondary antibodies. Blots were washed after primary and secondary antibodies with TBST (Tris-buffered saline plus 0.1% Tween 20) three times for 10 min. Western blots were quantified with the SynGene program (SynGene, Cambridge, UK) or the Image Lab Software (BioRad). Protein stability of Cse4 was measured as the percent remaining after normalization to Tub2 signal.

### Ubiquitination (Ub) pull-down assay

Ub pull-down assays for determining the levels of ubiquitinated Cse4 were performed as described previously (Au *et al.* 2013) with minor modifications. Strains were grown to logarithmic phase overnight in selective media, re-suspended in fresh media containing galactose/raffinose (2% final concentration each) and grown for four hours at 23°. Cells were resuspended in Cell Lysis Buffer with freshly added protease inhibitor cocktail, PMSF, and NEM (inhibitor for de-ubiquitination) and lysed by vortexing for 1 hr at 4° in the presence of glass beads. The concentration of proteins in each resulting lysate was measured and normalized. 50 µl lysate was saved for input and the remaining lysate was added to Tandem Ubiquitin Binding Entity (TUBE) beads (LifeSensors) and incubated overnight at 4°. Beads were centrifuged and washed three times with TBST on a rocking platform; unbound lysate was collected. Beads were resuspended in Laemmli buffer and incubated for 10 min at 100°. Input and unbound fraction containing Laemmli buffer were processed in parallel. Samples were analyzed using Western Blot. Western blots were quantified with the SynGene program (SynGene, Cambridge, UK). *p*-value was determined using a paired *t*-test (GraphPad Prism).

### Chromosome spreads

Chromosome spreads were performed as previously described (Collins *et al.* 2004; Crotti and Basrai 2004; Collins *et al.* 2007) with minor modifications. Cultures were grown to logarithmic phase in selective medium containing 2% raffinose and treated with Nocodazole (20 µg/ mL final) for three hours to arrest cells in the G2/M phase of the cell cycle. FACS analysis confirmed the cell cycle arrest. For the last hour of the Nocodazole arrest, galactose was added to 2% final concentration. Cells were lysed gently by treatment with zymolase-100T and BME. Spheroplasts were then spread onto glass slides and fixed with paraformaldehyde and 1% lipsol and allowed to air dry. Slides were washed with 1 X PBS for 10 min and incubated in 16B12 Mouse anti-HA primary antibody (1:2500). Slides were washed three times with 1 X PBS for 10 min and incubated with Cy3 conjugated Goat anti-mouse secondary antibody (1:5000). Slides were washed with 1 X PBS and mounted with antifade containing DAPI and visualized using DeltaVision Microscopy Imaging Systems. Nuclei with a single or two HA- or Flag-Cse4 foci were counted as normal Cse4 localization and nuclei with multiple foci or a diffused signal in the nucleus were counted as mislocalized Cse4. At least 360 cells were counted for each experiment. *p*-values were determined using Ordinary one-way ANOVA (GraphPad Prism).

### ChIP-seq

Chromatin immunoprecipitations were performed as previously described (Cole *et al.* 2014; Chereji *et al.* 2017) with modifications. Cultures grown to logarithmic phase in glucose or raffinose/galactose media for 1.75 hr were treated with formaldehyde (final 1%) for 20 min at 30° followed by the addition of 2.5 M glycine for 10 min. Cells were washed twice with 1 X PBS and resuspended in 2 mL FA Lysis Buffer (1 mM EDTA pH 8.0, 50 mM HEPES-KOH pH 7.5, 140 mM NaCl, 0.1% sodium deoxycholate, 1% Triton X-100) with 1 × protease inhibitors (Sigma) and 1 mM PMSF final concentration. The cell suspension was split into four screw top tubes with glass beads (0.4-0.65 mm diameter) and lysed three times for 40 sec each, followed by a five-minute rest on ice, and lysed two times for 40 sec each in an MP Bio FastPrep-24 5G. The cell lysate was collected, and the chromatin pellet was washed twice in FA Lysis Buffer. Each pellet was resuspended in 600 µl of FA Lysis Buffer and combined into one 5 ml tube. The chromatin suspension was sonicated 24 times with repeated 15 sec on/off at 20% amplitude using a Branson digital sonifer. After 3 min of centrifugation (13000 rpm, 4°), the supernatant was transferred to another tube. About 5% was used for input and checking the size of sheared DNA. The remaining was incubated with 150 µl anti-FLAG M2 Affinity Gel (Sigma, A2220-5ML) at 4C overnight. The beads were washed for five minutes on a rotator in 1 ml FA, FA-HS (500 mM NaCl), RIPA, and TE buffers twice each. The beads were resuspended in ChIP Elution Buffer (25 mM Tris-HCl pH 7.6, 100 mMNaCl, 0.5% SDS) and incubated at 65° overnight. The beads were treated with proteinase K (0.5 mg/ml) at 55° for four hours followed by Phenol/Chloroform extraction and ethanol precipitation. The DNA pellet was resuspended in a total of 50 µl sterile water.

Input and IP samples were repaired using the NEB Pre-PCR Repair Mix (New England Biolabs, M0309). Paired-end libraries for input and IP samples were prepared using the NEBNext End Prep (New England Biolabs, E7370) and NEBNext Multiplex Oligos for Illumina (New England Biolabs, E7335). Agencourt AMPure XP beads (Beckman-Coulter, A63880) were used to purify adaptor-ligated DNA samples and PCR products (input adapters diluted 1/3 and IP 1/250). The 50-base paired-end Illumina reads were aligned to the *S. cerevisiae* S288C reference (R64-2-1) using Bowtie version 1.0.0 with command line options -n2 -m1 -X 500. Duplicate reads (20–89%) were removed using Samtools rmdup (version 0.1.19). Between 1.4M and 5.3M unique alignments remained for the ChIP libraries and 14M-24M for the input libraries. The input alignments were randomly down sampled to 10M alignments each. Peaks were called using MACS (Zhang *et al.* 2008) version 2.1.1.20160226 in paired-end mode with default parameters and no additional down sampling.

The annotatePeaks tool of the Hypergeometric Optimization of Motif EnRichment suite (HOMER v5.10; http://homer.ucsd.edu/homer/) was used to assign peaks of Cse4 enrichment to genomic features. Customized annotations were utilized. Similar to the approach of Hildebrand and Biggins (Hildebrand and Biggins 2016), 5′- and 3′-UTR’s were annotated using the data of Nagalakshmi *et al.* (Nagalakshmi *et al.* 2008) downloaded from the yeast genome browser (https://browse.yeastgenome.org). 5′- and 3′-prime UTR data were available for 4605 and 5175 genes, respectively. For genes lacking UTR data, UTR’s were assigned a median length (53 and 105 nucleotides, respectively). Promoters were defined as the region lying 500 bp upstream of the transcription start site (*i.e.*, position 1 of the 5′-UTR). Transcription termination sites were defined as ± 50 bp from the end of the 3′-UTR.

Intersections between peak sets were computed using the IntersectRegions function of the USeq suite (http://useq.sourceforge.net) which also provides an estimate of statistical significance by randomization of one of the target peak sets across the genome. Coverage tracks were computed by MACS and normalized to 1M reads and displayed using the Integrative Genomics Viewer (Robinson *et al.* 2011).

### Data availability

Strains and plasmids are available upon request. Supplemental file S1 contains Table S1, which describes the strains used in this study, and Table S2, which lists the plasmids used. Figures S1, S2, and S3 are available as supplemental files. ChIP-seq data for wild type and *cdc7**-7* strains with *Flag-**Cse4* expressed from its own promoter and *GAL-Flag-**Cse4* integrated into the genome are available at GEO with accession number GSE148068. Supplemental material available at figshare: https://doi.org/10.25387/g3.12116610.

## Results

### Mutants of the Cdc7-Dbf4 kinase complex exhibit SDL with GALCSE4

**Figure 1:**
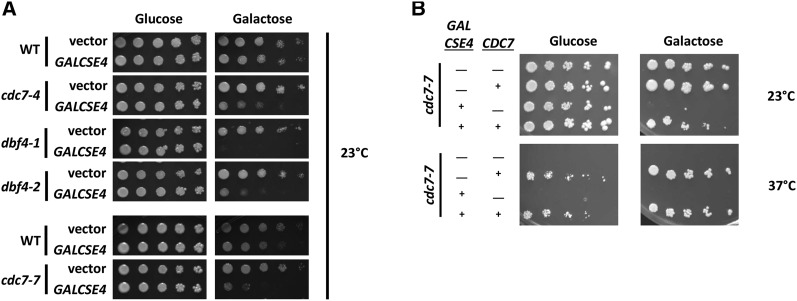
DDK mutants exhibit synthetic dosage lethality (SDL) to *GALCSE4*. A. Validation of *GALCSE4* SDL in *cdc7* and *dbf4* strains. Growth assays were done with wild type [BY4741 (for *cdc7**-4*, *dbf4**-1*, and *dbf4**-2*) and RSY299 (for *cdc7**-7*)], *cdc7**-4* (tsa131), *dbf4**-1* (tsa161), *dbf4**-2* (tsa162), and *cdc7**-7* (RSY302) strains transformed with vector (pMB433, vector) or *GAL-HA-**CSE4* (SB878, *GALCSE4*). Cells were spotted in fivefold serial dilutions on medium selective for the plasmid containing either glucose (2%, Cse4 expression off) or raffinose/galactose (2% each, Cse4 expression is on) and incubated at 23° for 3-5 days. Two independent transformants of *dbf4**-1*, *dbf4**-2*, and *cdc7**-4* strains and three independent transformants of *cdc7**-7* strains were assayed and a representative image is shown. B. The *GALCSE4* SDL phenotype of a *cdc7**-7* strain is linked to the *cdc7* mutant allele. Growth assays were done with *cdc7**-7* strains (RSY302 with pMB433 and RSY302 with pMB1597) transformed with empty vector (pRS425) or plasmid-born *CDC7* (pMB1898). Cells were spotted in fivefold serial dilutions on medium selective for the plasmids with glucose (2%) or raffinose/galactose (2% each). Plates were incubated at the indicated temperature for 5-7 days. Three independent transformants for each strain were assayed and a representative image is shown.

To identify mutants of essential genes that display synthetic dosage lethality (SDL) when Cse4 is overexpressed (*GALCSE4*), we performed a Synthetic Genetic Array (SGA) (Au *et al.* 2020). A strain in which *GAL-HA-**CSE4* was integrated in the genome was mated to an array of 786 conditional temperature sensitive strains. Growth at 26° of the haploid meiotic progeny was scored on galactose plates and the *p*-value was determined as previously described (Baryshnikova *et al.* 2010; Costanzo *et al.* 2010; Costanzo *et al.* 2016). Among the top twelve hits that show SDL are five alleles of genes encoding the Dbf4-dependent kinase (DDK) complex, the gene encoding calmodulin, and regulators of proteasome assembly, mRNA polyadenylation, and cell cycle progression (Table 1). The identification of multiple alleles encoding components of the DDK complex led us to further investigate a possible role of DDK in regulating cellular levels of Cse4 to prevent mislocalization of Cse4 to non-centromeric regions. We confirmed the SDL phenotype using growth assays in which yeast strains transformed with a plasmid containing *GALCSE4* or empty vector (vector) were plated on media with glucose or galactose to induce expression of *GALCSE4*. Strains with mutations in either *CDC7* (Figure 1A; *cdc7**-4*) or *DBF4* (Figure 1A; *dbf4**-1*, *dbf4**-2*) exhibited *GALCSE4* SDL at the permissive temperature of 23° on galactose media. A *cdc7**-7* mutant, which was not included in the SGA screen, also exhibited *GALCSE4* SDL (Figure 1A). We pursued in-depth studies with the *cdc7**-7* mutant because the *cdc7**-7* allele displays a stronger SDL phenotype at 23°, has a low frequency of induced mutagenesis, does not have defects in the cell cycle at 23°, and exhibits DNA replication defects only at the non-permissive temperature of 37° (Hollingsworth *et al.* 1992). To establish that the SDL phenotype of a *cdc7**-7 GALCSE4* strain is linked to the *CDC7* gene, we performed growth assays with *cdc7**-7 GALCSE4* strains with plasmid-borne *CDC7* or empty vector. The plasmid-borne *CDC7* rescued the temperature sensitivity of the *cdc7**-7* strain at 37° and the SDL phenotype of *cdc7**-7 GALCSE4* at 23° (Figure 1B).

**Table 1:** Twelve mutant alleles with the lowest score from a Synthetic Genetic Array (SGA) with temperature sensitive gene mutants overexpressing *CSE4*. Listed are the top twelve conditional alleles of essential genes that displayed SDL when *CSE4* is expressed from a galactose-inducible promoter (Au *et al.* 2020). Shown are the mutant allele, SGA score as the epsilon value calculated as previously in (Costanzo *et al.* 2010; Costanzo *et al.* 2016) with a negative value indicating a defect in growth, human ortholog (https://yeastmine.yeastgenome.org/yeastmine), and gene ontology (GO) annotation (https://www.yeastgenome.org/).

	Mutant	SGA score	Human ortholog	GO Category
**1**	***cdc7-4***	**-1.348**	***CDC7***	**DNA-dependent DNA replication initiation**
**2**	***dbf4-2***	**-1.22**	***DBF4***	**DNA-dependent DNA replication initiation**
**3**	***dbf4-ts***	**-1.206**	***DBF4***	**DNA-dependent DNA replication initiation**
**4**	***gpi12-ph***	**-1.13**	***PIGL***	**GPI anchor biosynthetic process**
**5**	***cdc23-1***	**-1.113**	***CDC23***	**Regulation of mitotic metaphase/anaphase transition**
**6**	***cmd1-1***	**-1.031**	***CALML3/5***	**Phosphatidylinositol biosynthetic process**
**7**	***dbf4-1***	**-0.989**	***DBF4***	**DNA-dependent DNA replication initiation**
**8**	***sts1-ph***	**-0.975**		**Proteasome localization**
**9**	***hrp1-1***	**-0.94**	***HNRNPA2B1***	**mRNA polyadenylation**
**10**	***rna15-58***	**-0.937**		**mRNA polyadenylation**
**11**	***cdc7-1***	**-0.927**	***CDC7***	**DNA-dependent DNA replication initiation**
**12**	***pre2-75***	**-0.916**	***PSMB11***	**Proteasome core complex assembly**

### Cdc7 regulates ubiquitin-mediated proteolysis of Cse4

**Figure 2:**
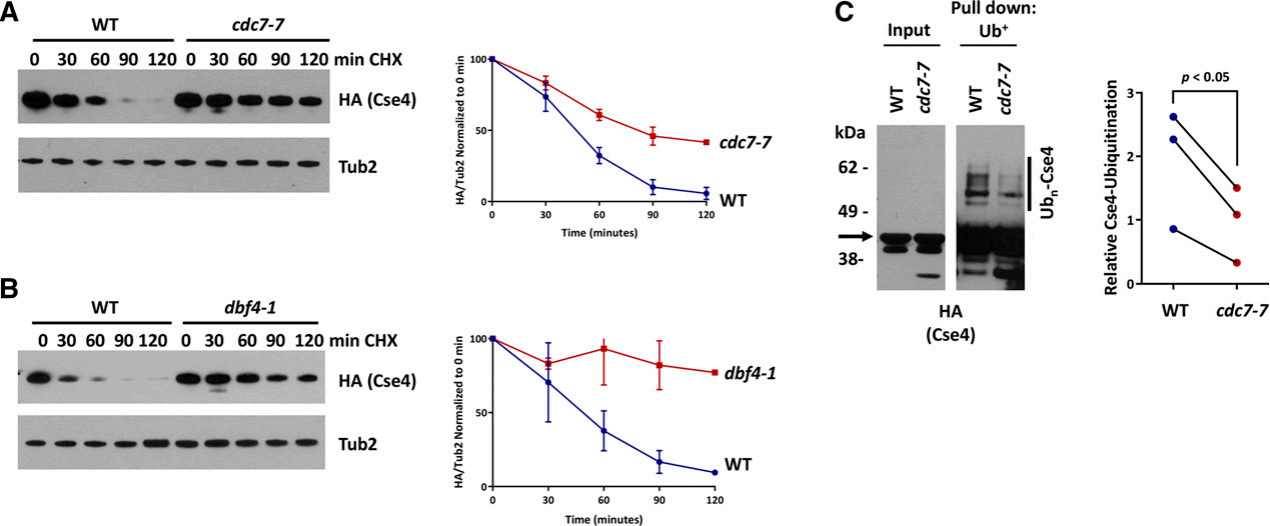
Cdc7 regulates ubiquitin-mediated proteolysis of Cse4. A. Cse4 is stabilized in a *cdc7* strain and B. Cse4 is stabilized in a *dbf4* strain. Western blot analysis of protein extracts prepared from wild type (BY4741 for *dbf4**-1* and RSY299 for *cdc7**-7*), (A) *cdc7**-7* (RSY302), and (B) *dbf4**-1* (TSA161) strains transformed with *GAL-HA-**CSE4* (pMB1597). Strains were grown to logarithmic phase of growth in raffinose-containing media (2%), and expression of *GAL-HA-**CSE4* was induced with galactose (2%) for four hours. Cells were then treated with cycloheximide (CHX, 10 µg/ml) and glucose (2%). Aliquots were taken at the indicated timepoints. Protein extracts were analyzed using Western blot analysis and blots were probed with anti-HA (Cse4) and anti-Tub2 (loading control). Quantification of the levels of HA-Cse4 remaining after treatment with CHX relative to Tub2 from two independent experiments is shown in the graphs. Error bars represent SEM. C. Ubiquitination of Cse4 is decreased in a *cdc7* strain. Ub-pull down assays were performed using protein extracts from wild type and *cdc7**-7* strains as described above and lysates were incubated with Tandem Ubiquitin Binding Entity beads (LifeSensors). Input and ubiquitin-enriched (Pull down: Ub^+^) samples were analyzed via Western blot against HA (left). Arrow indicates the unmodified Cse4 band. Quantification of levels of poly-ubiquitinated Cse4 (Ub_n_-Cse4) normalized to the levels in the input from three independent experiments is shown in the graph, *p*-value < 0.05.

Previous studies have shown that defects in ubiquitin-mediated proteolysis of overexpressed Cse4 contribute to *GALCSE4* SDL in *psh1**∆*, *slx5**∆*, and *hir2**∆* strains (Hewawasam *et al.* 2010; Ranjitkar *et al.* 2010; Ohkuni *et al.* 2016; Ciftci-Yilmaz *et al.* 2018). The SDL phenotype of DDK mutants led us to hypothesize that proteolysis of Cse4 is regulated by the DDK complex. Therefore, we examined the stability of overexpressed HA-Cse4 in wild type, *cdc7**-7*, and *dbf4**-1* strains after treatment with cycloheximide at 23°. Increased stability of HA-Cse4 was observed in *cdc7**-7* (Figure 2A) and *dbf4**-1* (Figure 2B) strains when compared to that in a wild type strain.

It has been shown that defects in ubiquitination of Cse4 contribute to increased protein stability and mislocalization of Cse4 in a *psh1**Δ* strain (Hewawasam *et al.* 2010; Ranjitkar *et al.* 2010). The increased stability of Cse4 led us to examine if a *cdc7**-7* strain exhibits defects in poly-ubiquitination of overexpressed HA-Cse4 (Ub_n_-Cse4). We performed an affinity pull-down of ubiquitinated proteins and consistent with previous studies (Au *et al.* 2013), we detected ubiquitinated Cse4 as a laddering pattern in wild type cells (Figure 2C). Quantification of signal intensities of Ub_n_-Cse4 normalized to signal intensities of Cse4 in input samples showed a significant reduction in the levels of ubiquitinated Cse4 in a *cdc7**-7* strain compared to a wild type strain (Figure 2C, *p*-value < 0.05). The defects in Cse4 proteolysis and Cse4 ubiquitination in the *cdc7**-7* strains suggest that Cdc7 regulates ubiquitin-mediated proteolysis of Cse4.

### Cdc7 regulates proteolysis of Cse4 independently of its role in DNA replication initiation

**Figure 3:**
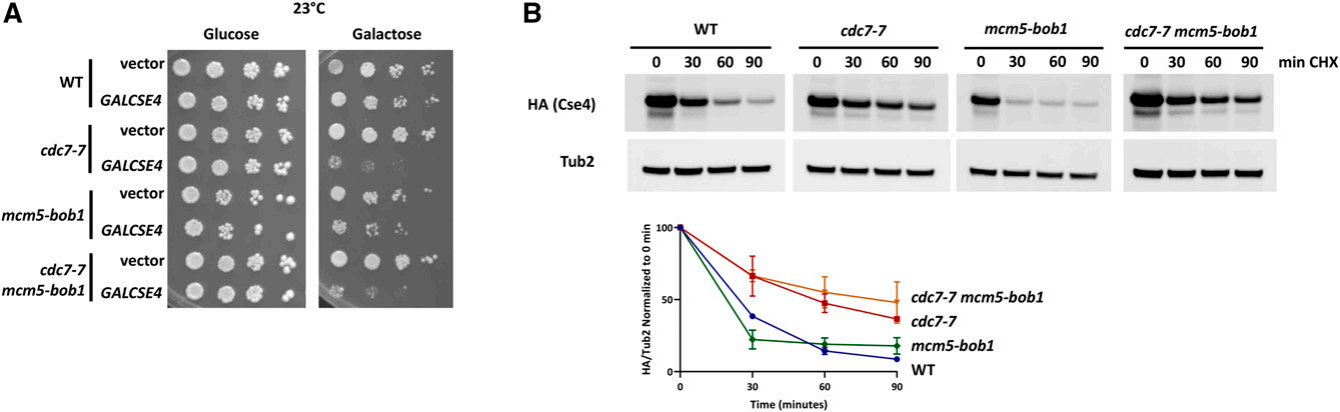
Cdc7 regulates stability of Cse4 independently of its role in initiation of DNA replication. A. A *cdc7**-7 **mcm5**-bob1* strain shows SDL with *GALCSE4*. Growth assays with wild type (RSY299), *mcm5**-bob1* (RSY867), *cdc7**-7* (RSY302), or *cdc7**-7 **mcm5**-bob1* (RSY847) strains transformed with vector (pMB433, vector) or *GAL-HA-**CSE4* (SB878, *GALCSE4*). Cells were spotted in fivefold serial dilutions on media selective for the plasmid containing either glucose (2%) or raffinose/galactose (2% each) and incubated at 23° for 3-5 days. Three independent transformants for each strain were assayed and the representative image is shown. B. A *cdc7**-7 **mcm5**-bob1* strain exhibits defects in Cse4 proteolysis. Western blot analysis of protein extracts from wild type (RSY299), *mcm5**-bob1* (RSY867), *cdc7**-7* (RSY302), or *cdc7**-7 **mcm5**-bob1* (RSY847) strains transformed with *GAL-HA-**CSE4* (pMB1597). Strains were grown to logarithmic phase of growth in raffinose-containing media (2%) and expression of *GAL-HA-**CSE4* was induced with galactose (2%) for four hours. Cells were then treated with cycloheximide (CHX, 10 µg/ml) and glucose (2%). Aliquots were taken at the indicated timepoints. Protein extracts were analyzed using Western blot analysis and blots were probed with anti-HA (Cse4) and anti-Tub2. (loading control). The graph shows the quantification of levels of HA-Cse4 remaining after treatment with CHX relative to Tub2 from two independent experiments. Error bars represent SEM.

Previous studies have shown that DDK activates the initiation of DNA replication through phosphorylation of the *MCM2*-7 complex (Lei *et al.* 1997; Oshiro *et al.* 1999; Weinreich and Stillman 1999; Zou and Stillman 2000; Bruck and Kaplan 2009). DNA replication defects are observed in *cdc7* strains at the non-permissive temperature of 37° (Sclafani 2000), but DNA replication is unperturbed in *cdc7**-7* strains grown at 23° (Jackson *et al.* 1993). Mutation of proline 83 of *MCM5* to leucine (*mcm5**-bob1*) bypasses specifically the requirement of Cdc7 for replication initiation and rescues the temperature sensitivity and replication defects of *cdc7**-1* and *cdc7**-7* strains at 37° (Hardy *et al.* 1997; Sclafani *et al.* 2002; Hoang *et al.* 2007). We observed the *GALCSE4* SDL phenotype and stability of HA-Cse4 in *cdc7**-7* strains at 23°. To further confirm that Cdc7-mediated proteolysis of Cse4 is independent of its role in initiating DNA replication, we performed growth assays for the SDL phenotype with the *cdc7**-7 **mcm5**-bob1* double mutant with *GALCSE4*. Our results showed that the *cdc7**-7 **mcm5**-bob1 GALCSE4* strain exhibited SDL similar to that observed in the *cdc7**-7 GALCSE4* strain at 23° (Figure 3A). Next, we determined if the *mcm5**-bob1* mutation affects the proteolysis of overexpressed HA-CSE4 in a *cdc7**-7* strain. Protein stability assays were done with extracts from wild type, *cdc7**-7*, *mcm5**-bob1*, and *cdc7**-7 **mcm5**-bob1* strains expressing *GAL-HA-**CSE4*. The stability of HA-Cse4 in the *mcm5**-bob1* strain was similar to that of the wild type strain (Figure 3B). Furthermore, the defects in proteolysis of HA-Cse4 observed in the *cdc7**-7* strain were not suppressed in the **cdc7*-7 **mcm5**-bob1* strain (Figure 3B). The inability of the *mcm5**-bob1* mutation to rescue the SDL phenotype and proteolysis defect in a *cdc7**-7 GALCSE4* strain suggests that the role of Cdc7 in regulating Cse4 proteolysis is independent of Cdc7’s role in initiating DNA replication.

### Cse4 is mislocalized to non-centromeric regions with an enrichment at promoters in a cdc7-7 strain

**Figure 4:**
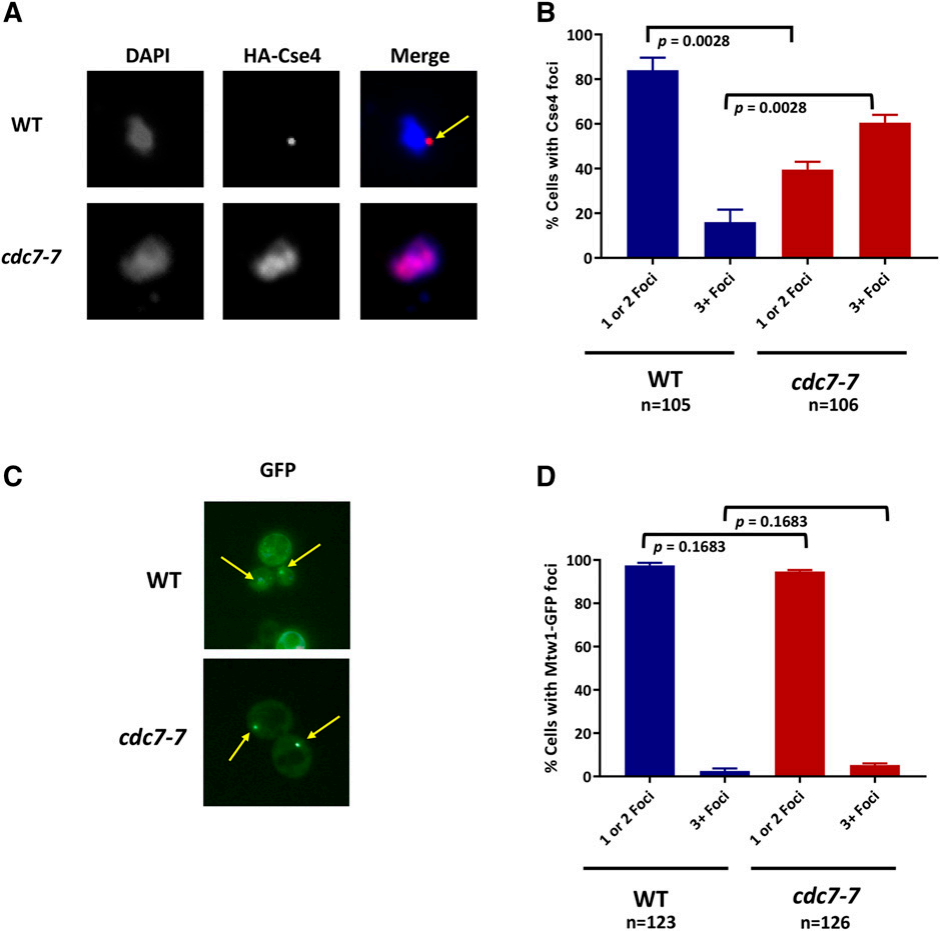
Cdc7 prevents mislocalization of Cse4 to non-centromeric regions. A. Cse4 is mislocalized in a *cdc7* strain. Localization of Cse4 was examined using chromosome spreads prepared from nocodazole arrested wild type (RSY299) and *cdc7**-7* (RSY302) strains transformed with *GAL-HA-**CSE4* (pMB1597). HA-Cse4 was labeled with Cy3 (red) and DNA with DAPI (blue). Representative images of cells showing normal localization counted as nuclei with one or two Cse4 foci (WT) and mislocalization counted as nuclei with more than two foci or a diffuse signal in the nucleus (*cdc7**-7*). Arrow indicates HA-Cse4 foci. B. Quantification of Cse4 localization from A. The graph displays the quantification of Cse4 localization as a percentage over total cell count. The SEM of two independent experiments is shown, WT 1 or 2 foci *vs.* *cdc7**-7* 1 or 2 Foci *p*-value = 0.0028; WT 3+ foci *vs.* *cdc7**-7* 3+ Foci *p*-value = 0.0028. C. The kinetochore protein *Mtw1* is not mislocalized in a *cdc7**-7* strain. Wild type (YMB9337) and *cdc7**-7* (YMB9338) cells were transformed with *Mtw1*-GFP on a plasmid (pMB1058), grown to logarithmic phase of growth, and analyzed for *Mtw1*-GFP (green) foci with live cell imaging. Representative images of cells showing single *Mtw1*-GFP foci are shown. Arrow indicates *Mtw1*-GFP foci. D. Quantification of *Mtw1*-GFP localization from C. The graph displays the quantification of cells with one or two GFP foci (normal) or with greater than three foci (mislocalized) with the SEM of two independent experiments; WT 1 or 2 foci *vs.* *cdc7**-7* 1 or 2 Foci *p*-value = 0.1683; WT 3+ foci *vs.* *cdc7**-7* 3+ Foci *p*-value = 0.1683.

We next examined the localization pattern of Cse4 using chromosome spreads, a method that eliminates soluble material to visualize chromatin-bound HA-Cse4 in WT and *cdc7**-7* strains. Previous studies have shown that Cse4 is localized to kinetochores that are clustered in one or two discrete nuclear foci in wild type cells, whereas mislocalization of Cse4 shows more than two foci or diffuse signal through the nuclear mass in *psh1**Δ*, *slx5**Δ*, and *hir2**Δ* strains (Hewawasam *et al.* 2010; Ranjitkar *et al.* 2010; Ohkuni *et al.* 2016; Ciftci-Yilmaz *et al.* 2018). In the *cdc7**-7* strain, we found that, in contrast to wild type cells, HA-Cse4 was mislocalized with signal at more than two foci or diffused across the nuclear mass (Figure 4A and 4B, *cdc7**-7*, *p*-value = 0.0028). To determine if the mislocalization of HA-Cse4 in a *cdc7**-7* strain is due to a kinetochore clustering defect, we examined the localization of the kinetochore protein *Mtw1*-GFP (Pinsky *et al.* 2003; Westermann *et al.* 2003). Our results showed a similar localization pattern of *Mtw1*-GFP to one or two foci in both the wild type (97.5%) and *cdc7**-7* (94.6%) cells (Figure 4C and 4D). This suggests that the mislocalization of HA-Cse4 in a *cdc7**-7* mutant is not due to kinetochore de-clustering. Based on these results, we conclude that DDK regulates ubiquitin-mediated proteolysis of Cse4 and prevents mislocalization of Cse4 to non-centromeric regions.

**Figure 5:**
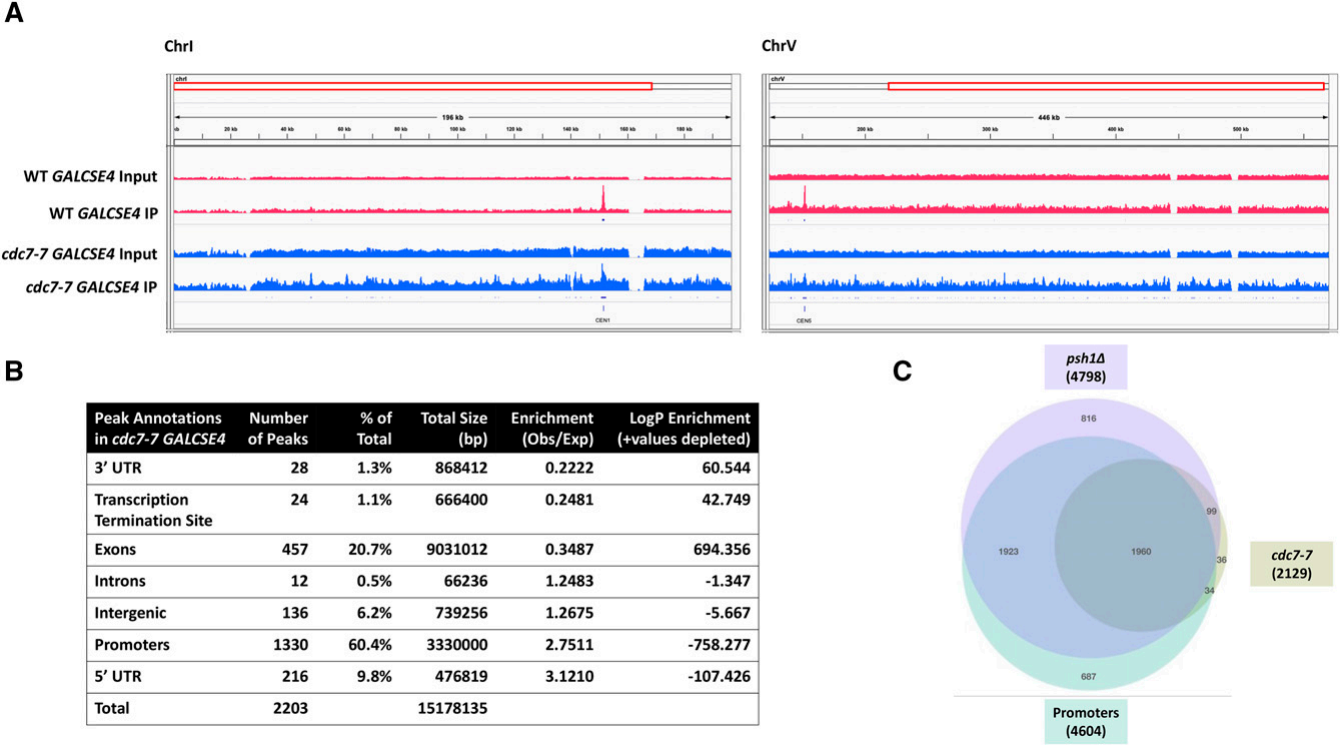
Cse4 is mislocalized to non-centromeric regions in a *cdc7**-7* strain. ChIP-seq was performed using chromatin lysates from wild type (YMB10044) and *cdc7**-7* (YMB10041) strains. A. Flag-Cse4 is mislocalized in a *cdc7**-7* strain. Genome browser of input and ChIP samples for Chromosome I and Chromosome V in wild type (top) and *cdc7**-7* (bottom) strains overexpressing Flag-Cse4. Regions of *CEN1* and *CEN5* are shown. B. Flag-Cse4 is enriched at promoters in a *cdc7**-7* strain. The annotatePeaks tool of HOMER v5.10 (http://homer.ucsd.edu/homer/) was used to define genomic locations of Flag-Cse4 enrichment in the *cdc7**-7* strain. The genomic feature, peak number, percent of total peaks, region size, fold-enrichment (relative to sequence content), and LogP enrichment are indicated. C. FLAG-Cse4 is preferentially enriched at promoters in *cdc7**-7* and *psh1**Δ* strains. Overlap between Flag-Cse4 enrichment in *cdc7**-7* and *psh1**Δ* strains and at promoters.

We next performed ChIP-seq experiments to define the genome-wide localization pattern of endogenous and overexpressed Cse4 in a *cdc7**-7* strain. ChIP-seq was performed using chromatin from wild type and *cdc7**-7* strains with endogenous Flag-Cse4 expressed from its own promoter grown at 23° in glucose or with galactose-inducible Flag-Cse4 integrated in the genome and grown at 23° in galactose media for 1.75 hr to overexpress Flag-Cse4. Consistent with previous reports (Hildebrand and Biggins 2016), endogenous Flag-Cse4 showed peaks of enrichment primarily at centromeric (*CEN*) regions in the wild type strain (Figure S1, WT). Endogenous Flag-Cse4 also showed enrichment primarily at *CEN* regions in the *cdc7**-7* strain (Figure S1, *cdc7**-7*), indicating that Flag-Cse4 expressed from its own promoter is not mislocalized to distinct non-centromeric genomic loci in a *cdc7**-7* strain. For overexpressed Flag-Cse4, at the sequencing depth of our experiments (1.5-5.3 million non-duplicates, uniquely-mapped reads), Flag-Cse4 was found enriched at only 30 non-*CEN* sites in a wild type strain. In contrast, 2,187 non-*CEN* peaks of Flag-Cse4 were detected in a *cdc7**-7 GAL-FLAG-**CSE4* strain. In addition, a higher generalized background of Flag-Cse4 was observed across the genome as evidenced by a lower signal to noise ratio of the *CEN* peaks (Figure 5A, S2). Our results show that overexpressed Flag-Cse4 is highly enriched at promoters (60.4% of total peaks and 2.75-fold enriched relative to feature target size), but not at 3′-UTR’s, transcription termination sites (TTS), exons, introns, and intergenic regions in the *cdc7**-7* strain (Figure 5B). Significant enrichment (3.1 fold) was also found at 5′-UTR’s, although only 9.8% of the total peaks were found at these locations. This result is likely attributable to peaks overlapping the boundary between promoter and 5′-UTR.

The phenotypes of SDL with *GALCSE4*, defects in Cse4 proteolysis, and mislocalization of Cse4 to non-centromeric regions in a *cdc7**-7* strain are similar to that observed in a *psh1**∆* strain (Hewawasam *et al.* 2010; Ranjitkar *et al.* 2010). Since the ChIP-seq experiments were performed with an isogenic set of strains with an integrated copy of *GAL-FLAG-**CSE4* in the same genetic background used previously to examine localization of overexpressed Flag-Cse4 in a *psh1**Δ* background (Hildebrand and Biggins 2016), we compared our results to the ChIP-seq results from Hildebrand and Biggins. The raw sequencing data were downloaded from the Sequence Read Archive (GEO Series GSE69696) and subjected to the same alignment and peak calling procedures used for our ChIP-seq analyses with the *cdc7**-7* strain. Of the 2,129 regions of Flag-Cse4 enrichment identified in the *cdc7**-7* strain 2,059 (97%) overlapped with one or more peaks of Cse4 enrichment identified in the *psh1**Δ* strain (*p*-value < 10^−4^) (Figure 5C). As observed for the *psh1**∆* strain, a high proportion (1,994/2,129, 94%) of Cse4 mislocalization in the *cdc7**-7* strain occurs in promoter regions (*p*-value < 10^−4^); virtually all were common to promoter-localized Cse4 found in the *psh1**Δ* strain (Figure 5C). We note that in making the 3-way comparison, closely-spaced peaks are merged to eliminate inconsistency in counts when a single peak in one set overlaps multiple peaks in another set; thus, the total number of intervals shown in Figure 5C differs from the actual *cdc7**-7* peak count indicated in Figure 5B. Overall, these results show that the mislocalization pattern of Cse4 in the *cdc7**-7* strain is similar to that observed in a *psh1**∆* strain.

### Cdc7 regulates Psh1-mediated proteolysis of overexpressed Cse4

**Figure 6:**
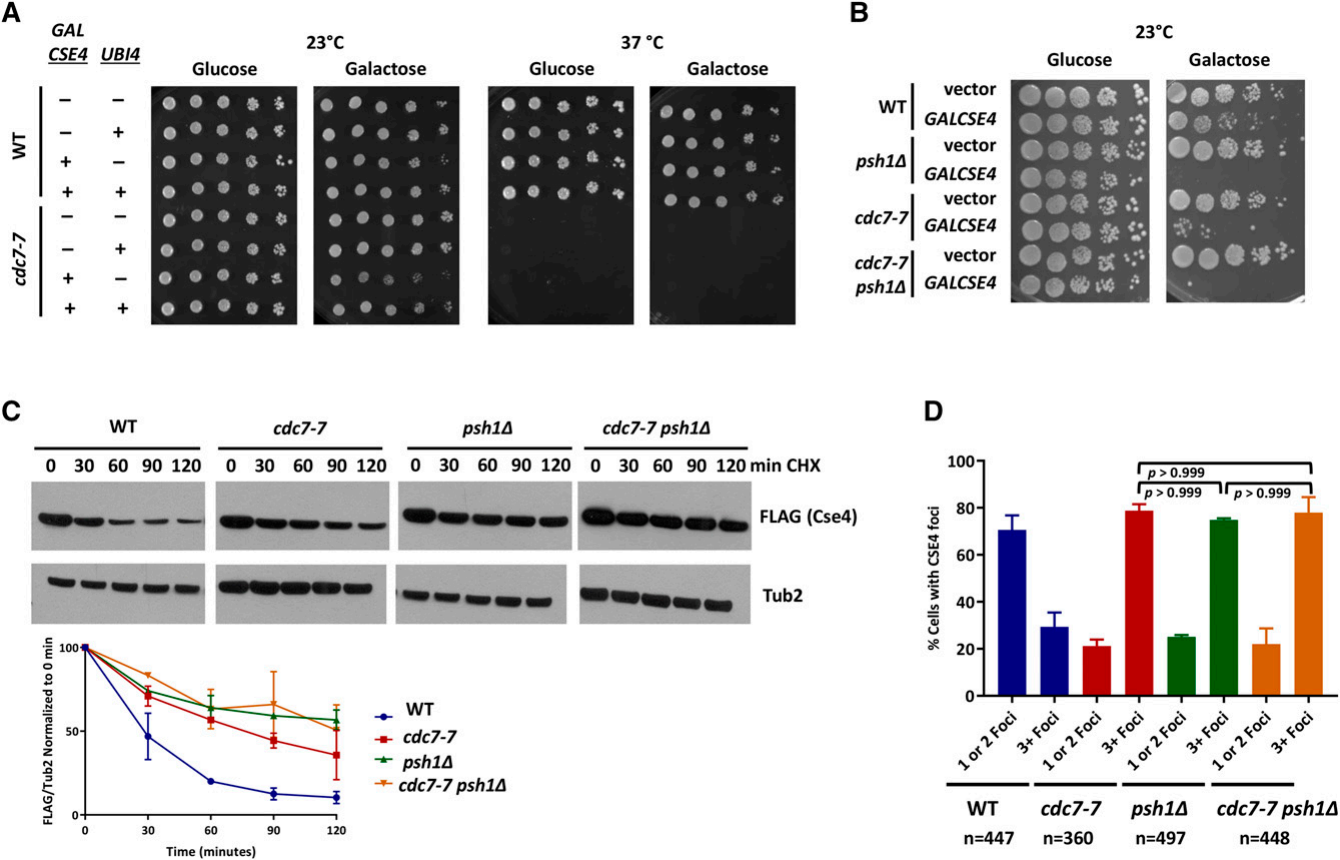
Cdc7 regulates Psh1-mediated proteolysis of Cse4. A. Overexpression of *UBI4* suppresses the SDL of a *cdc7**-7 GALCSE4* strain. Growth assays of wild type (RSY299) and *cdc7**-7* (RSY302) cells transformed with empty vector (pMB433, *GALCSE4* -) or *GAL-HA-**CSE4* (pMB1597, *GALCSE4+*) and subsequently transformed with empty (pRS425, 2µ *UBI4*-) or UBI4 (pMB1604, *UBI4*+). Cells were spotted in fivefold serial dilutions on media selective for the plasmids containing either glucose (2%) or raffinose/galactose (2% each) and incubated at 23° or 37° as indicated for 3-5 days. Three independent transformants for each strain were assayed and the representative image is shown. B. The *GALCSE4* SDL phenotype of the *cdc7**-7 **psh1**∆* strain is similar to that observed for *psh1**∆* and *cdc7**-7* strains. Growth assays of wild type, *psh1**∆*, *cdc7**-7*, and *cdc7**-7 **psh1**∆* strains with endogenously expressed Flag-Cse4 (vector; YMB10043, YMB10126, YMB10040, and YMB10124, respectively) or Flag-Cse4 expressed from a galactose-inducible promoter integrated into the genome (*GALCSE4*; YMB10044, YMB10127, YMB10041, and YMB10125, respectively) spotted in fivefold serial dilutions on to rich media containing either glucose (2%) or raffinose/galactose (2% each) and incubated at 23°C for 5 days. Three independent transformants for each strain were assayed and the representative image is shown. C. The Cse4 proteolysis defect in a *cdc7**-7 **psh1**∆* double mutant is similar to that observed for a *psh1**∆* strain. Western blot analysis of protein extracts from wild type (YMB10044), *cdc7**-7* (YMB10041), *psh1**∆* (YMB10127), and *cdc7**-7 **psh1**∆* (YMB10125) strains grown to logarithmic phase of growth in raffinose-containing media (2%). Expression of *GAL-FLAG-**CSE4* was induced with galactose (2%) for 1.75 hr. Cells were then treated with cycloheximide (CHX, 10 µg/ml) and glucose (2%). Aliquots were taken at the indicated timepoints. Protein extracts were analyzed using Western blot analysis and blots were probed with anti-FLAG (Cse4) and anti-Tub2 (loading control). The graph shows the quantification of the levels of FLAG-Cse4 remaining after treatment with CHX relative to Tub2 from two independent experiments. Error bars represent SEM. D. Mislocalization of Cse4 is not further enhanced in the *cdc7**-7 **psh1**∆* strain. Localization of Cse4 was examined using chromosome spreads prepared from nocodazole arrested wild type (YMB10044), *cdc7**-7* (YMB10041), *psh1**∆* (YMB10127), and *cdc7**-7 **psh1**∆* (YMB10125) strains. FLAG-Cse4 was labeled with Cy3 and DNA with DAPI. The graph displays quantification of Cse4 localization as a percentage over total cell count. The graph displays the SEM of two independent experiments, *psh1**Δ* 3+ foci *vs.* *cdc7**-7* 3+ Foci, *cdc7**-7* 3+ foci *vs.* *cdc7**-7 **psh1**Δ* 3+ Foci, and *psh1**Δ* 3+ foci *vs.* *cdc7**-7 **psh1**Δ* 3+ Foci *p*-value > 0.999.

We have previously shown that overexpression of the ubiquitin-encoding gene *UBI4* suppresses the SDL of a *psh1**Δ GALCSE4* strain (Au *et al.* 2013). The overlapping pattern of Cse4 mislocalization in *cdc7**-7* and *psh1**∆* strains prompted us to examine if overexpression of *UBI4* would suppress the SDL of a *cdc7**-7 GALCSE4* strain. Growth assays showed that *UBI4* suppresses the *cdc7**-7 GALCSE4* SDL phenotype at the permissive temperature of 23° (Figure 6A). *UBI4* did not suppress the TS growth defect of *cdc7**-7* strains at 37°.

We took multiple approaches to evaluate if Cdc7 functions in an overlapping pathway with Psh1 to regulate Cse4 proteolysis. We generated *cdc7**-7* and *psh1**∆* single and *cdc7**-7 **psh1**∆* double mutant strains with *GAL-FLAG-**CSE4* integrated in the genome. Growth assays confirmed the SDL phenotype of *GAL-FLAG-**CSE4* for *cdc7**-7* (Figure 1A and 6B), and that reported previously for *psh1**∆* strains on galactose media (Hewawasam *et al.* 2010; Ranjitkar *et al.* 2010). The *psh1**∆* strain showed a more severe growth defect than the *cdc7**-7* strain with *GAL-FLAG-**CSE4*. The *cdc7**-7 **psh1**∆* double mutant displays SDL similar to that observed for the *psh1**∆* strain (Figure 6B). We also examined the stability of overexpressed Flag-Cse4 in the *cdc7**-7*, *psh1**∆*, and *cdc7**-7 **psh1**∆* strains. Consistent with previous results (Figure 2A and (Hewawasam *et al.* 2010; Ranjitkar *et al.* 2010)), Flag-Cse4 was more stable in *cdc7**-7* and *psh1**∆* strains when compared to the wild type strain (Figure 6C). The stability of Flag-Cse4 in the *psh1**Δ* strain is higher than that in the *cdc7**-7* strain, however the stability of Flag-Cse4 in the *cdc7**-7 **psh1**∆* strain was similar to that observed in the *psh1**∆* strain (Figure 6C). Lastly, we examined the mislocalization of Flag-Cse4 in wild type, *cdc7**-7*, *psh1**∆*, and *cdc7**-7 **psh1**∆* strains using chromosome spreads. We observed significantly higher levels of Flag-Cse4 mislocalization in *cdc7**-7* as described earlier (Figures 4A and 4B) and as reported previously for *psh1**∆* strains (Hewawasam *et al.* 2010; Ranjitkar *et al.* 2010) when compared to the wild type strain. Consistent with results for the SDL phenotype and protein stability, the mislocalization of Flag-Cse4 was not further enhanced in *cdc7**-7 **psh1**∆* strains when compared to the single *cdc7**-7* and *psh1**∆* strains (Figure 6D, *p*-value > 0.999). We propose that Cdc7 and Psh1 are epistatic for proteolysis of Cse4 to prevent Cse4 mislocalization to non-centromeric regions.

## Discussion

In this study, we investigated the role of the Dbf4-dependent kinase (DDK) complex in proteolysis of Cse4. Five alleles of genes encoding DDK were among the top twelve hits in a screen to identify mutant strains displaying SDL with *GALCSE4*. Our results show that *cdc7**-7* strains exhibit an SDL phenotype with *GALCSE4*, defects in ubiquitin-mediated proteolysis of Cse4, and mislocalization of Cse4 to non-centromeric regions, particularly to gene promoters. The lack of a rescue of the *GALCSE4 SDL* or Cse4 proteolysis defect in the *cdc7**-7* strain by *mcm5**-bob1* indicates a DNA replication-independent role of Cdc7 in Cse4 proteolysis. Additionally, several experimental approaches showed that Cdc7 functions in a pathway overlapping with Psh1 to promote proteolysis of Cse4 and prevent Cse4 mislocalization to non-centromeric regions. Our studies define the first essential kinase, DDK, to regulate proteolysis of overexpressed Cse4 and prevent mislocalization of Cse4.

DDK is most well-studied for its role in initiating DNA replication through phosphorylation of the *MCM2*-7 DNA helicase complex at origins of replication, allowing cells to proceed through the G1/S phase of the cell cycle (Lei *et al.* 1997; Oshiro *et al.* 1999; Weinreich and Stillman 1999; Zou and Stillman 2000; Bruck and Kaplan 2009). Temperature sensitive *cdc7* mutants exhibit defects in the cell cycle and are unable to complete DNA replication at the restrictive temperature of 37°; replication and cell cycle defects are not observed at the permissive temperature of 23° (reviewed in (Sclafani 2000)). All the assays in our current study, including growth, protein stability, and chromosome localization, were performed at 23°. Based on these results, we conclude that the *GALCSE4* SDL phenotype, defect in Cse4 proteolysis, and decrease in Ub_n_-Cse4 levels in *cdc7**-7* strains observed at 23° are independent of defects in cell cycle progression.

Phosphorylation of *MCM2*-7 by DDK causes a conformational change in the *MCM2*-7 complex and this regulates replication initiation (Hoang *et al.* 2007). A mutation in MCM5, P83L (*mcm5**-bob1*), is thought to mimic the conformational change that results from DDK-mediated phosphorylation of *MCM2*-7. The *mcm5**-bob1* mutation rescues the temperature sensitivity, bypasses the cell cycle defects of *cdc7* strains (Jackson *et al.* 1993), and the DNA distribution by FACS of a *cdc7* *mcm5**-bob1* strain is normal (Hardy *et al.* 1997). We used genetic and biochemical approaches to examine if the role of Cdc7 in proteolysis of Cse4 is independent of its role in replication initiation. We reasoned that if the regulation of Cse4 proteolysis by Cdc7 was dependent on replication initiation, the *mcm5**-bob1* mutation should rescue the SDL phenotype and Cse4 proteolysis defect in *cdc7**-7* strains. However, we did not observe suppression of the *GALCSE4* SDL or defects in Cse4 proteolysis in the *cdc7**-7 **mcm5**-bob1* strain at 23°. Furthermore, ChIP-seq using a *cdc7**-7* strain did not reveal a significant enrichment of Cse4 to origins of DNA replication which are normally occupied by Cdc7 (Rossbach *et al.* 2017). Similar to our observations, a previous study has shown that *mcm5**-bob1* cannot suppress the defect of *cdc7*-induced mutagenesis (Pessoa-Brandão and Sclafani 2004), indicating a different Cdc7 substrate in mutagenesis than the *MCM2*-7 complex (Rossbach and Sclafani 2016). Together, our results support a DNA replication-independent role of Cdc7 in regulating Cse4 proteolysis.

Cse4 expressed from its own promoter is not detectably mislocalized to specific genomic regions in a *cdc7**-7* strain (Figure S1). Additionally, degradation of endogenous Flag-Cse4 in a *cdc7**-7* strain is similar to that in a wild type strain (Figure S3). Genome-wide studies have shown that mislocalization of Cse4 is barely detectable in wild type (Camahort *et al.* 2009; Lefrancois *et al.* 2009; Hildebrand and Biggins 2016) or *psh1**Δ* strains (Hildebrand and Biggins 2016), suggesting that cellular levels of endogenous Cse4 are stringently regulated to ensure that it is not mislocalized to non-centromeric regions in a wild type cell. In the context of overexpressed Cse4, wild type cells do not show growth inhibition with *GALCSE4*, in part because overexpressed Cse4 is proteolyzed by Psh1, Rcy1, Slx5, Ubr1, and other regulators. Mutants of these regulators display defects in proteolysis of Cse4, which contributes to mislocalization of overexpressed Cse4 and lethality with *GALCSE4* (Hewawasam *et al.* 2010; Ranjitkar *et al.* 2010; Ohkuni *et al.* 2016; Ciftci-Yilmaz *et al.* 2018).

Our studies here provide evidence that Cdc7 plays a role in regulating levels of overexpressed Cse4. Chromosome spreads showed mislocalization of overexpressed Cse4 in a *cdc7**-7* strain and ChIP-seq confirmed these results. We observed a significant amount of Cse4 mislocalization throughout the genome, and analysis of the localization pattern showed a preferential enrichment of Flag-Cse4 at promoter regions with a high degree of overlap to that observed in the *psh1**Δ* strain. We propose that Cdc7 functions in a pathway that overlaps with Psh1 in Cse4 proteolysis. We provide several lines of evidence to support our hypothesis. The *GALCSE4* SDL phenotype, increased stability of Cse4, and levels of Cse4 mislocalization observed in the *cdc7**-7 **psh1**Δ* strain were not significantly different than that observed in the *cdc7**-7* or *psh1**∆* strains. Additionally, the preferential localization of Cse4 to promoters is observed in both *cdc7**-7* and *psh1**Δ* strains and the *GALCSE4* SDL phenotype is suppressed by overexpression of *UBI4* in both *cdc7**-7* and *psh1**∆* strains. Future studies will allow us to investigate the mechanism by which Cdc7 affects the Psh1 pathway and if Cdc7 regulates pathways other than Psh1-mediated proteolysis for Cse4.

Previous studies have shown that Cdc7 and Dbf4 associate with replication origins, including the early-firing replication origins at the centromere (Natsume *et al.* 2013; Rossbach *et al.* 2017) and that low levels of DDK at centromeres contributes to delay in the replication of centromeres (Natsume *et al.* 2013). DDK associates with kinetochores through the COMA complex, consisting of Ctf19, Mcm21, Okp1, and Ame1, and this regulates sister chromatid cohesion independently of the role of DDK in initiating DNA replication (Natsume *et al.* 2013). DDK phosphorylates the N-terminal tail of Ctf19 and this recruits the cohesin loader Scc2/4, for proper sister chromatid cohesion (Hinshaw *et al.* 2017). Recent studies have shown that the N-terminal tail of Cse4 interacts with Okp1, which directs kinetochore loading distinct from Mif2-directed loading (Fischböck-Halwachs *et al.* 2019). Phosphorylation of the N-terminal tail of Cse4 promotes the interaction of Cse4 with Ame1/Okp1 and this likely regulates recruitment of kinetochore components (Hinshaw and Harrison 2019). It is of great interest to examine if Cse4 is a substrate of DDK and define the role of DDK-mediated phosphorylation of Ctf19 for the association of the COMA complex with Cse4. Future studies will allow us to examine if DDK-mediated phosphorylation of kinetochore substrates such as Cse4, Psh1, and Ctf19 contribute to the proteolysis of overexpressed Cse4 and prevent its mislocalization to non-centromeric regions.

In this study, we have described a new role for the essential kinase Cdc7 in regulating Psh1-mediated proteolysis of Cse4 independently of Cdc7’s role in initiating DNA replication. Based on our results for SDL of *GALCSE4* in *cdc7**-7* strains, we propose that inhibition of Cdc7 in cancers with high levels of CENP-A would lead to cancer cell-specific cell death. These studies are relevant from a clinical standpoint because high levels of Cdc7 and Dbf4 expression have been reported in several types of cancers (Bonte *et al.* 2008) and this correlates with accelerated progression through the cell cycle, mutation of p53, resistance to DNA damaging agents and chemotherapy, and poor survival rates (Montagnoli *et al.* 2004; Bonte *et al.* 2008; Kulkarni *et al.* 2009; Rodriguez-Acebes *et al.* 2010; Hou *et al.* 2012; Cheng *et al.* 2013). Targeting Cdc7 through siRNA knockdown in cancer cells has been shown to result in cancer cell-specific apoptotic cell death (Bonte *et al.* 2008; Kulkarni *et al.* 2009; Hou *et al.* 2012), whereas non-cancerous cells arrest in G1 and resume proliferation after Cdc7 activity is restored (Rodriguez-Acebes *et al.* 2010). Currently, Cdc7 inhibitors are in clinical trials to downregulate Cdc7 activity in cancer cells (clinicaltrials.gov #’s NCT02699749, NCT03096054). The evolutionary conservation of CENP-A and DDK makes budding yeast an excellent model to investigate the molecular role of DDK in preventing mislocalization of CENP-A and CIN.
